# Prognostic Value of Perineural Invasion on Survival and Recurrence in Oral Squamous Cell Carcinoma

**DOI:** 10.3390/diagnostics12051062

**Published:** 2022-04-24

**Authors:** Steffen Spoerl, Silvia Spoerl, Stephanie Reil, Michael Gerken, Nils Ludwig, Juergen Taxis, René Fischer, Tobias Ettl, Torsten E. Reichert, Gerrit Spanier

**Affiliations:** 1 Department of Cranio-Maxillofacial Surgery, University Hospital Regensburg, 93053 Regensburg, Germany; steffen.spoerl@ukr.de (S.S.); stephi.reil@web.de (S.R.); nils.ludwig@ukr.de (N.L.); juergen.taxis@ukr.de (J.T.); tobias.ettl@ukr.de (T.E.); torsten.reichert@ukr.de (T.E.R.); 2 Department of Internal Medicine 5—Hematology/Oncology, Friedrich-Alexander University Erlangen-Nürnberg, 91054 Erlangen, Germany; silvia.spoerl@uk-erlangen.de; 3 Tumor Center, Institute for Quality Management and Health Services Research, University of Regensburg, 93053 Regensburg, Germany; michael.gerken@ukr.de; 4 Department of Otorhinolaryngology, University Hospital Regensburg, 93053 Regensburg, Germany; rene.fischer@ukr.de

**Keywords:** oral squamous cell carcinoma, oral cancer, PNI, perineural invasion, survival, recurrence

## Abstract

A diagnosis of perineural invasion (PNI) is widely accepted as an unfavorable prognostic factor in various solid malignancies. Although PNI has been described as a high-risk parameter in oral squamous cell carcinoma (OSCC), its role in the current staging manuals of the American Joint Committee on Cancer (AJCC) is rather subordinate. We analysed the prognostic value of PNI on survival and recurrence in a large, multicenter OSCC cohort and a population-based approach. A total of 493 OSCC patients with primary tumor resection to negative margins and concomitant neck dissection between 2010 and 2017 were enrolled. PNI was evaluated in relation to overall survival (OAS) and recurrence-free survival (RFS) using uni- and multi-variable Cox regression. The median follow-up time was 5.0 years and PNI was diagnosed in 48 patients (9.7%). A pathohistological verification of PNI correlated significantly with a deteriorated OAS in uni- (HR 2.312; 95% CI 2.312–3.493, *p* = 0.001) and multivariable Cox regression (HR 1.820; 95% CI 1.164–2.847, *p* = 0.009). Additionally, a diagnosis of PNI correlated with increased cumulative, as well as distant, metastasis 5-year-recurrence rates (*p* = 0.027 and *p* = 0.011, respectively). The application of adjuvant radiotherapy (RT) or radiochemotherapy (RCT) in patients with PNI did not alter OAS or RFS in survival analysis when compared to patients without PNI. The results underline the adverse impact of PNI on the survival and recurrence of surgically treated OSCC patients. Based on our findings, we highly recommend an emphasis on PNI in the TNM staging concept.

## 1. Introduction

Despite the tremendous efforts of the recent decade aiming to ameliorate the outcome of patients suffering from oral squamous cell carcinoma (OSCC), survival rates are still regrettably low [1]. Certainly, the clinical application of immunotherapeutic approaches in the recent years has resulted in promising courses of disease—at least for a selected proportion of OSCC patients [2]. Among the many oncological hallmarks of solid malignancies, and particularly with regards to OSCC, metastasis and invasive growth are major challenges for surgical treatment [3]. Tumor size, anatomical site, and infiltration pattern determine the involvement and invasion of locoregional nerves due to a high anatomical density of these structures in the oral cavity and in the head and neck region, in general. For a tumor entity explicitly characterized as neurotropic [4,5], it is not surprising that perineural invasion (PNI) is commonly observed in OSCC [3]. When it comes to assessing outcomes in OSCC patients with a pathohistological verification of PNI, survival was fundamentally decreased in comparison to patients with an absence of PNI [6].

Since the first description of PNI by Cruveilhier in 1835 [7], numerous authors have characterized the phenomenon of tumor cells invading adjacent structures. The overall tenor of most studies explicitly considers tumors with pathohistological verified PNI as more aggressive, with a consecutively higher chance of adverse patient outcomes [8,9]. Although the exact molecular and cellular mechanisms of PNI are not entirely clear, recent studies have focused on the microenvironment closely located to nerval structures. In the classic concept, PNI was considered as a mainly cancer-driven nerve infiltration directly through the path of least resistance in the perineural space. This process includes a broad orchestra of different cell types and soluble, as well as non-soluble, factors [10,11,12]. However, more recent findings suggest that PNI requires reciprocal signaling interactions between tumor cells and nerve components, particularly Schwann cells. Specifically, OSCC can express neurotrophins and neurotrophin receptors that may contribute to cancer cell migration towards nerves, PNI, and neuritogenesis towards cancer. Schwann cells may play an important role in promoting PNI by migrating towards cancer cells, intercalating, and dispersing cancer, and facilitating cancer migration towards nerves [12]

Although PNI was correlated with various adverse cancer properties [4], its precise impact on prognosis in OSCC is not entirely clear [4]. Additionally, this topic is particularly relevant for the indication of adjuvant treatment modalities. We therefore evaluated the role of PNI in OSCC patients, focusing on therapeutic implications.

## 2. Materials and Methods

### 2.1. Patient Selection

This retrospective, multicenter cohort study comprises patients with newly diagnosed OSCC and surgical treatment between January 2010 and December 2017. All participants were residing in the region of Eastern Bavaria, which represents a German population of around 2.3 million people, and includes the districts of Upper Palatinate and Lower Bavaria. The population-based dataset was kindly provided by the Clinical Cancer Registry of the Tumor Center Regensburg. Diagnostic workup and treatment took place at three different centers: the Department of Cranio-Maxillofacial Surgery, as well as the Department of Otorhinolaryngology, both at the University Hospital Regensburg, and the Department of Otorhinolaryngology at the St. Elisabeth Hospital Straubing.

All included patients received resection of the primary lesion to negative margins based on clinical and radiologic examination. The standard surgical approach was transoral. In cases where this technique did not offer adequate exposure, temporary mandibulotomy and pull-through resection were applied. No robotic surgery was used in the three above-mentioned centers. In addition to the different types of oral defect reconstruction, a diligent elective/selective neck dissection was performed in each patient. Previous neck dissection, primary radio(-chemo)therapy of head and neck squamous cell carcinoma, or neoadjuvant treatment modalities were the exclusion criteria.

Consecutively, 493 patients were included in the present study. Staging was carried out in line with the “TNM classification of malignant tumors” by the *Union Internationale Contre le Cancer* (UICC) in its 7th edition [13]. Adjuvant treatment was based on the recommendation of the multidisciplinary tumor board, and radiotherapy or radio-chemotherapy was used accordingly. Patient-specific demographic, pathohistological, and clinical data were obtained from medical records, and included gender, age at diagnosis, positive anamnesis of nicotine and alcohol abuse, anatomical site, extranodal spread, grading, and application of adjuvant therapies. With consideration of comorbidities and age on survival of OSCC patients, the age-adjusted Charlson Comorbidity Index (ACCI) was calculated as previously described and without taking OSCC into account [14,15]. Recurrent disease was either diagnosed by radiologic evidence with clinical correlation or histologic confirmation by biopsy. Survival follow up data concerning recurrence-free survival (RFS) and overall survival (OAS) were gathered from medical records, death certificates, registration offices, and the Clinical Cancer Registry of the Tumor Center—Institute for Quality Management and Health Services Research, University of Regensburg. PNI was diagnosed if routine pathohistological examination of primary surgical specimen by an experienced pathologist showed a tumor in ultimate proximity to a nerve which is surrounded by the malignancy by at least one third circumference or penetrates at least one of three connective tissue layers of the nerve [16]. The cut-off date for this study was set at 31 March 2021.

### 2.2. Statistics

Continuous data are described as means, median, minimum, and maximum values. Categorical data are given as absolute frequencies and relative percentages. Patient characteristics were compared using the two-tailed Student’s t test for continuous data, in the case of normal distribution; otherwise, the Mann–Whitney U test was applied. Pearson’s Chi-square test was used for testing independence between categorical variables.

Recurrences were derived from clinical reports and were analyzed in detail, where local, locoregional relapse, as well as formation of distant metastases, were evaluated. Five -year-survival rates for OAS and RFS, as well as cumulative recurrence rates, were analyzed from the date of resection until the first event. Patients’ OAS and RFS were calculated with the Kaplan–Meier method. The follow-up period and survival times were right censored using 31 March 2021 as the cut-off date, rendering a median follow-up of 5.0 years (4.7 years for the PNI-group). Differences in survival outcomes were tested for statistical significance by the two-sided log rank test (Mantel–Cox), and the level of significance was set at 0.05. Besides univariable survival analysis, we additionally performed multivariable Cox regression to adjust for covariables.

Before setting up a multivariable model, multicollinearity among prognostic variables was checked by using the variance inflation factor (VIF), which measures the correlation between the predictor variables in a linear regression model. Besides the pathohistological diagnosis of PNI, we additionally adjusted for gender, ACCI, history of smoking and alcohol abuse, distinct tumor localizations, UICC stage, grading, lymph vessel invasion, and vein invasion. The variables were consecutively incorporated in multivariable analyses if *p*-values of univariable analysis were less than 0.100.

ACCI was categorized in three groups, reflecting increased age at diagnosis and comorbidities. Hazard ratios (HR) and corresponding 95% confidence intervals (CI) were estimated and considered statistically significant when the CI excluded 1.0, and a two-sided *p*-value was <0.05. All analyses were performed by using IBM SPSS Statistics, version 26.0 (IBM Corp., SPSS for Windows, Armonk, NY, USA).

## 3. Results

Here, we report on the results of a retrospective multicenter cohort study on 493 OSCC patients who had resection to negative margins combined with an elective neck dissection. The prevalence of PNI at the time of diagnosis was 9.7% (48 patients). Patient characteristics of the entire retrospective cohort, including demographic and clinical parameters, are provided in Table 1. Most patients were male (70.6%), and most patients had an age of between 50 and 69.9 years at the date of tumor resection (66.9%). Most patients displayed an ACCI of >0 (91.7%), with 240 patients (48.7%) presenting an ACCI of ≥3. The majority of patients additionally presented a history of alcohol abuse (67.7%), as well as nicotine abuse (73.8%). The predominant tumor localizations were the floor of mouth (36.7%), as well as the tongue (30.0%). The majority of tumors were staged as pT1 (38.7%) and pT2 (31.8); however, 190 patients (38.6%) had already presented cervical lymph node metastasis at the day of tumor resection (pN+). In summary, UICC stage IV could be diagnosed in 187 patients (37.9%). In 42.2% of patients, an adjuvant treatment was applied.

**Figure 1:**
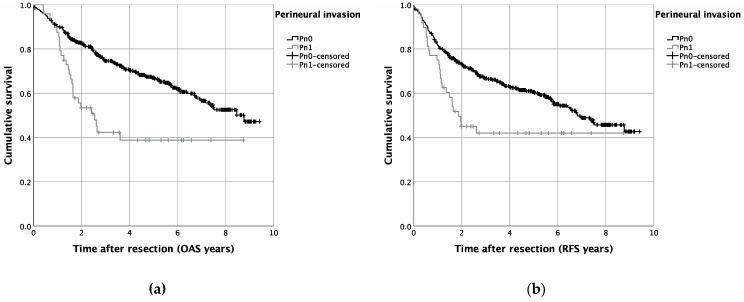
Survival in OSCC patients with or without PNI (Pn1 and Pn0, respectively): Kaplan–Meier curves for OAS (**a**) (*p* = 0.001) and RFS (**b**) (*p* = 0.002).

**Figure 2:**
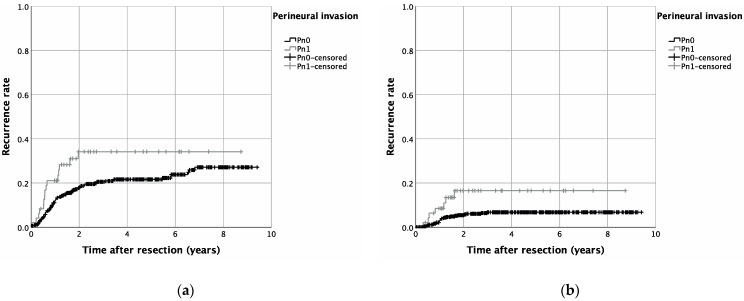
Recurrence rates in OSCC patients with or without PNI (Pn1 and Pn0, respectively) for cumulative recurrence (**a**) (*p* = 0.001) as well as for recurrence by distant metastasis (**b**) (*p* = 0.002).

PNI was significantly associated with higher T-stages (*p* = 0.002), cervical lymph node involvement (*p* < 0.001), and pathohistological verification of extranodal spread (*p* < 0.001). Additionally, lymph vessel invasion (*p* < 0.001), as well as vascular invasion (*p* = 0.016), were more prevalent in patients with PNI (Pn1). Pearson’s chi-squared test identified increasing UICC stages to be significantly associated with PNI (*p* < 0.001) (Table 1). Univariable survival analysis revealed a significantly reduced OAS, as well as RFS, for patients with pathohistological verified PNI: hereby, a Kaplan–Meier analysis resulted in a five year OAS of 67.0% for patients without PNI (Pn0), whereas in case of PNI, five year survival was reduced to 38.8% (*p* < 0.001) (Figure 1). Similar results were seen for RFS, with a five year RFS of 60.6% (Pn1) and 42.0% (Pn1) (*p* = 0.004) (Figure 1). A cumulative five-year-recurrence rate of 21.6% (Pn0) and 34.2% (Pn1) was observed (*p* = 0.027) (Table 2, Figure 2). When focusing on recurrence in detail, a diagnosis of PNI did not significantly alter local (*p* = 0.134), as well as locoregional, recurrence rates (*p* = 0.303). A five-year distant metastasis recurrence rate, however, was significantly increased for patients diagnosed with PNI (16.6%) when compared to Pn0 patients (6.8%) (*p* = 0.011) (Table 2, Figure 2).

Multivariable survival analysis was additionally performed to adjust for covariables such as gender, ACCI, smoking and alcohol consumption, anatomical tumor site, UICC stage, grading, and lymphatic and vascular invasion. For OAS, a pathohistological verification of PNI significantly impaired the survival of OSCC patients in the present multicenter cohort study (HR 1.820; 95% CI 1.164–2.847, *p* = 0.009) (Table 3). Additionally, an ACCI of 3+ (HR 1.877; 95% CI 1.028–3.430, *p* = 0.040), as well as advanced tumor stages (HR 2.741; 95% CI 1.954–3.845, *p* < 0.001), were associated with diminished OAS (Table 4). For RFS, however, merely a trend in impaired multivariable survival was observed for PNI in OSCC patients (HR 1.461; 95% CI 0.934–2.284, *p* = 0.097) (Table 4). In line with the results of the multivariable analysis for OAS, UICC stages III and IV (HR 2.074; 95% CI 1.534–2.805, *p* < 0.001) and an elevated ACCI (*p* < 0.001) were significantly associated with impaired RFS (Table 4).

## 4. Discussion

PNI is a so-called optional descriptor in the established “TNM Classification of Malignant Tumours, 8th Edition” for OSCC, in addition to lymphatic and vascular invasion. Cancerous invasive growth in adjacent anatomical structures is characteristically linked to an increased risk of local, cervical lymphatic, and distant disease recurrence [17,18]. Nonetheless, it seems like PNI is relatively neglected in existing staging manuals [19]. The importance on OSCC patients’ survival after curative tumor resection is particularly of high clinical relevance.

Among the special features of our study are the large cohort, the population-based approach, the differentiation between types of recurrence, and the focus on patients’ comorbidities. With 9.7% of OSCC patients being diagnosed with PNI in the present study, a comparison with the prevalence of PNI in similar study designs might be of interest: a broad bandwidth (6.1–82%) for prevalence of PNI in OSCC can be found in the literature [20]. It is important to note that the diagnosis of PNI is closely related to different clinical, as well as pathohistological, parameters, including comorbidities, higher UICC stage, and heterogeneity of patient cohorts [21], especially blurring in the definition of PNI, as well as explicit pathohistological examination methods, to determine the evidence of PNI in clinical routine and scientific approaches.

A commonly applied definition of PNI entails the presence of malignant cells within any of the three layers of a nerval structure or tumor foci outside of the nerve with involvement of more than one third of the nerve’s circumference [16]. A less precise definition in this regard was given by Batsakis in 1985: PNI can be diagnosed if “tumor cell invasion in, around and through the nerve…” is present [22]. Undoubtedly, the definition of Batsakis leaves a scope for interpretation when it comes to precise definition of PNI. Therefore, objective, broadly accepted parameters for the diagnosis of PNI are urgently needed [23]. In addition, the establishment of standardized protocols would improve diagnoses of PNI. Certainly, immunohistochemical staining enhances the reliable detection of PNI based on neuronal markers such as S100 and several neurotrophins and their receptors [12,24]. In this regard, Shen et al. conducted a study on 116 previously resected OSCC patients, re-evaluating diagnoses of PNI by pathohistological stainings [25]. The initial PNI rate was increased by 17% through sheer review of hematoxylin and eosin-stained tissue biopsies by experienced pathologists. Additionally, IHC staining was applied using anti-S100 antibodies, which resulted in an overall PNI detection rate of 51% [25].

A major aim of the present study was to evaluate PNI as a prognostic oncological factor of surgically treated OSCC patients. Our results underline the findings of previous cohort studies on OSCC patients, indicating a significantly impaired outcome for Pn1 OSCC patients in uni- as well as multivariable survival analyses [6,26,27]. Moreover, with the present retrospective cohort study, we investigated whether PNI in OSCC patients affects different types of recurrence after tumor resection. Kaplan–Meier analysis clearly indicates a pronounced risk for recurrence, whether in terms of overall recurrence or recurrence by formation of distant metastasis. In this regard, we were able to attribute OSCC patients with PNI a 1.8-fold increased risk to develop distant metastasis, mainly in the lung or liver. This should be considered in follow-up care and assessment of OSCC patients.

However, the significance of PNI with regards to recurrence of malignancy is controversial in the current literature—notably, for the risk of developing distant metastasis, a diverging opinion exists. In this regard, Tarsitano et al. published results of not significantly increased distant metastasis recurrence rates for Pn1 patients compared to Pn0 OSCC cases [27]. In contrast to this, Rahima et al. attributed increased distant metastasis recurrence rates to oral, as well as oropharyngeal, squamous cell carcinoma patients with PNI; however, just 101 patients were included in this retrospective cohort study [28].

The results from our population-based, multicenter cohort study corroborate the findings of Rahima et. al. [28], and thereby assign PNI as a clearly detrimental prognostic impact on survival and recurrence in OSCC patients. In terms of predictive implications of PNI, and, chiefly, for adjuvant treatment modalities, we could not derive reliable implications of a potential outcome benefit after adjuvant RT or RCT due to small case numbers. Unsurprisingly, this question is of growing interest in the current literature. A main argument, potentially explaining a lack of effect for application of adjuvant treatment in Pn1 patients, might potentially be based on an already deteriorated outcome of Pn1 patients when compared to Pn0 cases. Particularly with regard to promising in vitro findings [11], the current inconsistent data situation regarding the role of adjuvant treatment of OSCC patients with PNI definitely demands further research [29].

## 5. Conclusions

PNI appears to be a relevant prognostic factor for the survival and recurrence of OSCC patients having received tumor resection to negative margins combined with a cervical lymphadenectomy. When focusing on recurrence in detail, the development of distant metastasis was significantly increased in Pn1 patients. Currently, an indication of adjuvant treatment for verified PNI in OSCC patients is under debate. So far, the present multicenter cohort study could not derive reliable implications of a potential outcome benefit after adjuvant RT or RCT. Additional in vivo, as well as in vitro, studies are necessary to unveil the molecular basis of PNI in OSCC and to develop targeted therapeutical approaches to address this special tropism of tumor spread. Based on our findings, we highly recommend emphasizing PNI in the TNM staging concept.

## Figures and Tables

**Table 1:** Clinicopathological characteristics of OSCC patients according to the diagnosis of PNI (*n* = 445).

Category Group		Perineural Invasion						
		Pn0			Pn1		Total	χ^2^
		N	(%)	N	(%)	N	(%)	*p*
Gender	Female	133	29.9%	12	25.0%	145	29.4%	0.480
	Male	312	70.1%	36	75.0%	348	70.6%	
Age at diagnosis	<50	48	10.8%	5	10.4%	53	10.8%	0.983
	50.0–59.9	156	35.1%	17	35.4%	173	35.1%	
	60.0–69.9	143	32.1%	14	29.2%	157	31.8%	
	70.0–79.9	77	17.3%	9	18.8%	86	17.4%	
	80.0+	21	4.7%	3	6.3%	24	4.9%	
Age-adjusted CCI	0	36	8.1%	5	10.4%	41	8.3%	0.724
	1 and 2	190	42.7%	22	45.8%	212	43.0%	
	3+	219	49.2%	21	43.8%	240	48.7%	
Positive anmnesis smoking	No	119	26.7%	10	20.8%	129	26.2%	0.376
	Yes	326	73.3%	38	79.2%	364	73.8%	
Positive anmnesis alcohol	No	146	32.8%	13	27.1%	159	32.3%	0.420
	Yes	299	67.2%	35	72.9%	334	67.7%	
Anatomical tumor site	Buccal mucosa	33	7.4%	1	2.1%	34	6.9%	0.332
	Upper alveolus and gingiva	19	4.3%	1	2.1%	20	4.1%	
	Lower alveolus and gingiva	71	16.0%	5	10.4%	76	15.4%	
	Hard palate	32	7.2%	2	4.2%	34	6.9%	
	Tongue	132	29.7%	16	33.3%	148	30.0%	
	Floor of mouth	158	35.5%	23	47.9%	181	36.7%	
Tumor size	T1	183	41.1%	8	16.7%	191	38.7%	0.002
	T2	141	31.7%	16	33.3%	157	31.8%	
	T3	37	8.3%	9	18.8%	46	9.3%	
	T4	84	18.9%	15	31.3%	99	20.1%	
Cervical lymph node metastasis	N0	286	64.3%	17	35.4%	303	61.5%	<0.001
	N1	60	13.5%	6	12.5%	66	13.4%	
	N2/3	99	22.2%	25	52.1%	124	25.2%	
Extranodal spread	No	108	24.3%	18	37.5%	126	25.6%	<0.001
	Yes	50	11.2%	13	27.1%	63	12.8%	
	Not applicable	287	64.5%	17	35.4%	304	61.7%	
Grading	G1	35	7.9%	1	2.1%	36	7.3%	0.192
	G2	324	72.8%	34	70.8%	358	72.6%	
	G3/4	86	19.3%	13	27.1%	99	20.1%	
Lymphatic invasion	L0	399	89.7%	27	56.3%	426	86.4%	<0.001
	L1	46	10.3%	21	43.8%	67	13.6%	
Vascular invasion	V0	435	97.8%	44	91.7%	479	97.2%	0.016
	V1	10	2.2%	4	8.3%	14	2.8%	
UICC stage	I	144	32.4%	3	6.3%	147	29.8%	<0.001
	II	78	17.5%	8	16.7%	86	17.4%	
	III	69	15.5%	4	8.3%	73	14.8%	
	IV	154	34.6%	33	68.8%	187	37.9%	
Adjuvant therapy	No	277	62.2%	8	16.7%	285	57.8%	<0.001
	Radiotherapy	109	24.5%	24	50.0%	133	27.0%	
	Radio-chemotherapy	59	13.3%	16	33.3%	75	15.2%	
Life status	Alive	295	66.3%	21	43.8%	316	64.1%	0.002
	Deceased	150	33.7%	27	56.3%	177	35.9%	
Death/recurrence	Alive without recurrence	263	59.1%	21	43.8%	284	57.6%	
	Death or recurrence	182	40.9%	27	56.3%	209	42.4%	0.041
	Total	445	100.0%	48	100.0%	493	100.0%	

Abbreviations: CCI: Charlson Comorbidity Index; UICC: Union Internationale Contre le Cancer.

**Table 2:** Cumulative recurrence rates of as well as detailed forms of recurrence in OSCC patients, depending on PNI.

Category	Group	N (Included Patients)	N (Recurrent Disease)	5-Year-Recurrence Rate	Log-Rank *p*
Cumulative recurrence rate	Pn0 + Pn1	493	106		0.027
	Pn0	445	91	21.6%	
	Pn1	48	15	34.2%	
Local recurrence rate	Pn0 + Pn1	493	67		0.134
	Pn0	445	58	13.8%	
	Pn1	48	9	21.1%	
Locoregional recurrence rate	Pn0 + Pn1	493	48		0.303
	Pn0	445	42	10.5%	
	Pn1	48	6	17.1%	
Distant metastasis recurrence rate	Pn0 + Pn1	493	33		0.011
	Pn0	445	26	6.8%	
	Pn1	48	7	16.6%	

**Table 3:** Effect of PNI and additional covariables on OAS using uni- as well as multivariable Cox regression.

Overall Survival (OAS)									
Category	Group	Univariable Cox Regression				Multivariable Cox Regression			
		*p*	HR	Lower 95%-CI	Upper 95%-CI	*p*	HR	Lower 95%-CI	Upper 95%-CI
PNI	Pn0		1.000				1.000		
	Pn1	<0.001	2.312	1.530	3.493	0.009	1.820	1.164	2.847
Gender	Female		1.000						
	Male	0.857	1.030	0.746	1.424				
Age-adjusted CCI	0	<0.001	1.000			<0.001	1.000		
	1 and 2	0.778	0.915	0.491	1.704	0.774	0.912	0.488	1.707
	3+	0.043	1.854	1.020	3.369	0.040	1.877	1.028	3.430
Positive anamnesis smoking	No		1.000						
	Yes	0.522	1.118	0.795	1.572				
Positive anamnesis alcohol	No		1.000						
	Yes	0.684	1.068	0.777	1.469				
Anatomical tumor site	Upper alveolus, gingiva and hard palate	0.23	1.000						
	Tongue	0.126	0.665	0.395	1.121				
	Lower alveolus, floor of mouth, buccal mucosa	0.499	0.851	0.534	1.358				
UICC stage	I/II		1.000				1.000		
	III/IV	<0.001	3.015	2.168	4.194	<0.001	2.741	1.954	3.845
Grading	G1/2		1.000				1.000		
	G3/4	0.068	1.395	0.976	1.993	0.124	1.328	0.925	1.906
Lymphatic invasion	L0		1.000				1.000		
	L1	0.001	1.906	1.297	2.800	0.595	1.122	0.735	1.713
Vascular invasion	V0		1.000						
	V1	0.036	2.055	1.049	4.027	0.185	1.583	0.803	3.119

Abbreviations: CCI: Charlson Comorbidity Index; UICC: Union Internationale Contre le Cancer.

**Table 4:** Effect of PNI and additional covariables on cumulative RFS using uni- as well as multivariable Cox regression.

Recurrence-Free Survival (RFS)									
Category	Group	Univariable Cox Regression				Multivariable Cox Regression			
		*p*	HR	Lower 95%-CI	Upper 95%-CI	*p*	HR	Lower 95%-CI	Upper 95%-CI
PNI	Pn0		1.000				1.000		
	Pn1	0.005	1.794	1.195	2.692	0.097	1.461	0.934	2.284
Gender	Female		1.000						
	Male	0.894	1.020	0.758	1.373				
Age-adjusted CCI	0	<0.001	1.000			<0.001	1.000		
	1 and 2	0.743	0.911	0.523	1.589	0.578	0.852	0.486	1.496
	3+	0.070	1.642	0.960	2.808	0.108	1.561	0.907	2.688
Positive anamnesis smoking	No		1.000						
	Yes	0.684	1.066	0.782	1.454				
Positive anamnesis alcohol	No		1.000						
	Yes	0.638	1.072	0.801	1.436				
Anatomical tumor site	Upper alveolus, gingiva and hard palate	0.028	1.000			0.290			
	Tongue	0.008	0.535	0.337	0.850	0.130	0.691	0.428	1.115
	Lower alveolus, floor of mouth, buccal mucosa	0.099	0.709	0.471	1.067	0.108	1.561	0.907	2.688
UICC stage	I/II		1.000						
	III/IV	<0.001	2.331	1.745	3.113	<0.001	2.074	1.534	2.805
Grading	G1/2		1.000				1.000		
	G3/4	0.058	1.375	0.989	1.910	0.136	1.289	0.923	1.800
Lymphatic invasion	L0		1.000				1.000		
	L1	0.001	1.874	1.311	2.679	0.275	1.251	0.837	1.871
Vascular invasion	V0		1.000						
	V1	0.161	1.614	0.826	3.152				

Abbreviations: CCI: Charlson Comorbidity Index; UICC: Union Internationale Contre le Cancer.

## Data Availability

Data can be obtained by scientists that conducted the work independently from the industry on request. Data are not stored on publicly available servers.
